# Bacteriological profile of intra-abdominal infections in a tertiary care hospital

**Published:** 2018-08

**Authors:** Sukanya Sudhaharan, Padmaja Kanne, Lakshmi Vemu, Padmasri Chavali, Shanker Rao Desmukha, Bheerappa Nagari

**Affiliations:** Nizam’s Institute of Medical Sciences Panjagutta, Hyderabad, Telangana, India

**Keywords:** Pancreatic necrosis, Perforation, Cholecystitis, Appendicitis

## Abstract

**Background and Objectives:**

Intra-abdominal infections (IAIs) include a wide spectrum of pathological conditions, ranging from uncomplicated appendicitis to fecal peritonitis .The resulting infections should be diagnosed early and treated based on the organism isolated and its susceptibility. In this study the bacteriological profile and antimicrobial resistance pattern of intra-abdominal infections was analyzed.

**Materials and Methods:**

A retrospective analysis of samples received from 119 cases of intra-abdominal infections in our Institute from January 2015 to December 2017 was analyzed. Patients with primary peritonitis from cirrhosis or ascites were not included in the study. The specimens were primarily processed, as per standard methods. Identification and antimicrobial susceptibility testing was done by the Vitek-2 system. Anaerobic culture was performed on 5% sheep blood agar plates and incubated in GEN bag anaerobic pouches.

**Results:**

In our study perforative peritonitis 43/119 (36.1%) was predominant IAI followed by acute pancreatitis 14/119 (11.7%) and pancreatic necrosis 12/119 (10%). Microbial growth was observed in 66.3% (79/119) of the cases and combined infections were observed in14/119 (11.7%) of the cases. *Escherichia coli* was the predominant organism isolated in 58/119 (40.8%), out of which 41/58 (70.6%) were ESBL producers and 16/58 (27.5%) were multi drug resistant isolates. *Klebsiella pneumoniae* was isolated from 11/119 (9.2%) cases out of which 8/11 (72.7%) were ESBL and 3/11 (27.2%) were multidrug resistant isolates. Post-operative complications was observed in 12/119 (10%) patients with mortality in 15/119 (12.6%) patients.

**Conclusion:**

Early diagnosis and appropriate management of the infections will help to prevent the morbidity and mortality associated with these infections.

## INTRODUCTION

Intra-abdominal infections (IAIs) include a wide spectrum of pathological conditions, ranging from uncomplicated appendicitis to fecal peritonitis (1). The resulting infection is typically polymicrobial and comprised of both aerobic and anaerobic microbes, which need systemic antimicrobial therapy (2). Antibiotic therapy has to be modified based on culture and susceptibility testing. Surgery along with appropriate antibiotics will help to reduce the morbidity and mortality associated with intra-abdominal infections.

Aim & objectives of this study were: 1) To study the bacteriological profile and antimicrobial resistance pattern of the organisms isolated from IAI and 2) To study the type of IAI, organ involved and mortality associated with IAI.

## MATERIALS AND METHODS

A retrospective analysis of samples received from 119 cases of intra-abdominal infections undergoing surgery or interventional drainage in our institute from January 2015 to December 2017 was analyzed. Patients with primary peritonitis from cirrhosis or ascites were not included in the study.

The specimens such as peritoneal fluids, pus, bile, ascitic fluid received to the microbiology department were primarily processed, as per standard methods, on 5% sheep blood agar (aerobic and anaerobic) and Chromogenic agar (CPS ID, bioMerieux, Marcy Elitoile, France) and also inoculated into Bact/Alert SN bottles. Identification and antimicrobial susceptibility testing was done by the Vitek-2 system (bio-Merieux, Marcy Elitoile, France). Anaerobic culture was performed on 5% sheep blood agar plates and incubated in GEN bag anaerobic pouches (bioMerieux, Marcy l’Etoile-France).

## RESULTS

**Fig. 1.**
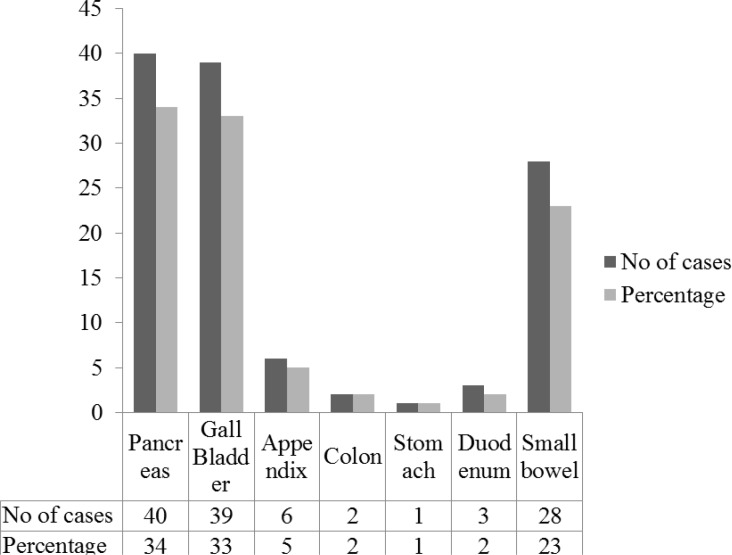
Source of infection according to organ of origin

In our study perforative peritonitis, 43/119 (36.1%), was predominant infection followed by acute pancreatitis 14/119 (11.7%) and pancreatic necrosis 12/119 (10%). Other infections include cholecystitis, cholelithiasis, choledocholithiasis (Table 1). Perforations of abdominal organs include small bowel, appendix, gall bladder, stomach, duodenum and colon. (Fig. 1). Small bowel is the common organ perforated in 28/43 (65.1%) of the cases.

**Table 1.** Total No of cases (n=119)

**Infection**	**Sterile**	**Monomicrobial infections**	**Polymicrobial infections**	**Total**
Acute pancreatitis	6	8	0	14
Chronic pancreatitis	2	2	1	5
Pancreatic necrosis	3	5	4	12
Pancreatic pseudocyst	4	5	0	9
Appendicular abscess	2	2	0	4
Acute cholecystitis	2	0	0	2
Cholangitis	1	7	1	9
Choledochal cyst	4	1	1	6
Choledocholithiasis	1	9	2	12
Cholelithiasis	2	1	0	3
Perforations (n=43)				
Appendix	2	0	0	2
Gall bladder	2	5	0	7
Colon	1	1	0	2
Stomach	1	0	0	1
Duodenum	1	2	0	3
Small bowel	6	17	5	28
Total IAI’S	40	65	14	119

As per organ type involved pancreas was the predominant organ involved in 40/119 (33.6%) cases (Table 2).

**Table 2.** Gram negative bacilli isolated

**Infection**	***Escherichia coli***	***Klebsiella pneumoniae***	***Proteus mirabilis***	***Pseudomonas aeruginosa***
Pancreas				
Acute pancreatitis	5	0	1	0
Chronic pancreatitis	2	0	1	0
Pancreatic necrosis	5	2	2	0
Pancreatic pseudocyst	2	0	0	0
Total	14	2	4	-
**Appendix**				
Appendicular abscess	2	0	0	0
**Gall bladder**				
Cholangitis	7	1	0	1
Choledochal cyst	2	0	0	0
Choledocholithiasis	8	2	0	0
Cholelithiasis	1	0	0	0
Total	18	3	-	1
**Perforation**				
Gall bladder perforation	4	1	0	0
Duodenal perforation	2	0	0	0
Colon perforation	0	1	0	0
Small bowel perforation	18	5	0	0
Total	24	7	-	-
TOTAL	58	12	4	1

Microbial growth was observed in 66.3% (79/119) of the patients and among them 14/119 (11.7%) were combined infections (Table 1). *E. coli* was the predominant organism isolated in 58/119 (40.8%) of IAI’s, of which 41/58 (70.6%) were ESBL producers and 16/58 (27.5%) were multi drug resistant isolates. *K. pneumoniae* was isolated from 11/119 (9.2%) cases of which 8/11 (72.7%) were ESBL and 3/11 (27.2%) were multidrug resistant isolates. Other organisms isolated include *K. pneumonia*, *Proteus mirabilis*, and *Pseudomonas aeruginosa* (Table 2).

Majority of the *E. coli* was isolated in cases with small bowel perforation 18/58 (31%) followed by choledocholithiasis 8/58 (13.7%) (Table 2). Other Gram negative and Gram positive organisms isolated in different IAI’s are shown in Tables 2–4. The antibiotic susceptibility pattern of the organisms is listed in Tables 5 and 6.

**Table 3.** Other Gram negative bacilli isolated

**Organisms**	**No of cases**	**Infection**
*Enterobacter cloacae*	2	Pancreatic Pseudocyst
		Small bowel perforation
*Stenotrophomonas*	3	Chronic pancreatitis
*S. maltophilia*		Acute pancreatitis
		Pancreatic necrosis
*Morganella morganii*	1	Pancreatic necrosis
*Aeromonas hydrophila*	2	Choledocholithiasis
*Burkholderia cepacia*	1	Pancreatic necrosis
*Citrobacter freundii*	1	Choledochal cyst
*Bacteroides*	2	Acute pancreatitis
		Pancreatic Necrosis

**Table 4.** No of Gram positive cocci isolated

**Infection**	**MRSA**	**MSSA**	***Enterococcus***
Chronic pancreatitis	1	0	0
Pancreatic pseudocyst	0	1	0
Choledocholithiasis	0	0	1
Small bowel perforation	0	0	3
Total	1	1	4

**Table 5.** Antibiotic susceptibility pattern of Gram negative isolates

**Organisms**	**No. of isolates**	**% Susceptibility to specified antibiotics**												

		**Amikacin**	**Gentamicin**	**Ciprofloxacin**	**Levofloxacin**	**Cotrimoxazole**	**Ceftazidime**	**Cefepime**	**Imipenem**	**Meropenem**	**Pip/TaZ**	**Sulb/cefape**	**Colistin**	**Tigecycline**
***Escherichia coli***	58	48.8	48.8	11.9	11.9	10.8	1.7	1.7	70.6	70.6	47	42.3	100	100
***Klebsiella pneumoniae***	12	45.7	45.7	12.9	12.9	11.4	0	0	72.7	72.7	14.3	21.4	81.4	80.9
***Proteus mirabilis***	4	75	75	50	50	75	0	0	50	100	100	100	IR	IR
***Pseudomonas aeruginosa***	1	100	0	0	0	IR	0	0	0	0	0	0	100	IR
***Enterobacter cloacae***	2	100	100	0	0	100	100	100	100	100	100	100	100	100
***Stenotrophomonas S. maltophilia***	3	IR	IR	IR	100	100	100	NT	IR	IR	IR	IR	NT	NT
***Morganella morganii***	1	100	100	0	0	0	100	100	100	100	100	100	IR	IR
***Aeromonas hydrophila***	2	100	100	100	100	100	50	50	100	100	50	100	100	NT
***Burkholderia cepacia***	1	IR	IR	IR	100	100	100	IR	IR	100	IR	IR	IR	IR

IR Intrinsically resistant

NT Not tested

**Table 6.** Antibiotic susceptibility pattern of Gram positive isolates

**Organisms**	**No. of Isolates**	**% Susceptibility to specified antibiotics**												

		**Penicillin**	**Oxacillin**	**Gentamicin**	**Ciprofloxacin**	**Levofloxacin**	**Co-t rimoxazole**	**Tetracycline**	**Clindamycin**	**Erythromycin**	**Linezolid**	**Vancomycin**	**Teicoplanin**	**Daptomycin**
MRSA	1	0	0	0	0	0	0	0	0	0	100	100	100	100
MSSA	1	0	100	100	0	0	100	100	100	100	100	100	100	100
***Enterococcus faecium***	4	0	IR	IR	0	0	IR	50	IR	0	100	100	100	100

IR Intrinsically resistant

NT Not tested

**Fig. 2.**
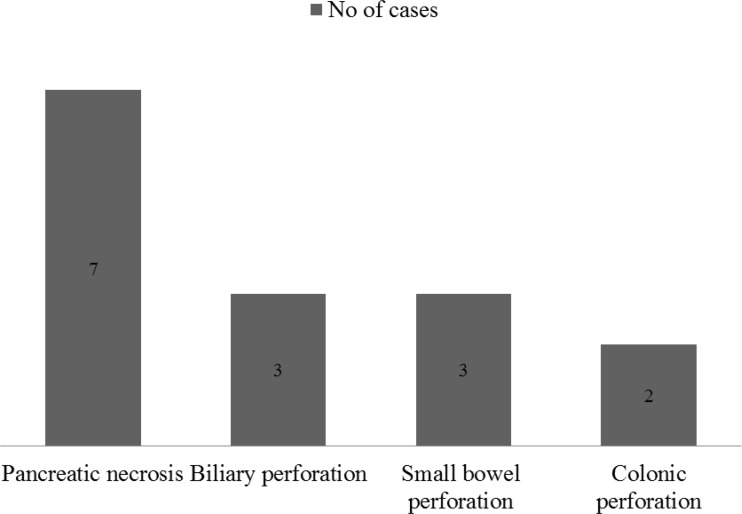
Mortality

Post-operative complications (anastomotic leak, pleural effusion, gastro-intestinal fistula, sepsis) was observed in 12/119 (10%) patients and mortality in 15/119 (12.6%) patients (Fig. 2).

## DISCUSSION

Intra-abdominal infection varies widely, ranging from peritonitis to intrahepatic infection to diverticulitis, appendicitis, and intra-abdominal abscess. IAI’s are common surgical emergencies and they are responsible for non-trauma deaths worldwide (3).

The pathogenesis of intra abdominal infections is determined by bacterial factors which influence the transition from contamination to infection. Bacterial activation leads to transmigration of granulocytes resulting in increased permeability, edema and protein rich peritoneal exudates (4).

Perforative peritonitis was seen in 36.1% of our cases. Perforation is one of the serious infections which require immediate surgery and both aerobic and anaerobic flora are isolated from peritoneal fluid (3). In a study from India about the spectrum of peritonitis, they found that gastroduodenal perforation is most common accounting for 54.2% of the cases (5) in contrast to our study. In a study from Serbia, they found that small bowel perforations accounted for 23% of the cases (6) whereas in our study it is the commonest of perforations accounting for 65.1%. Our patients were treated by surgery with closure of perforation & resection and anastomosis.

In a study by CIOWA (7) most of the cases of IAI are due to appendicitis (33.3%) followed by cholecystitis (14.6%) and small bowel perforation (7.6%). The involvement of appendix was low in our study indicating less complicated course of the disease requiring referral to a tertiary care centre. Reshetnyak et al. found that in Western countries, gallstone disease in men was 7.9% and it was 16.6% in women (8) whereas Sun et al. found in Asia, it ranged 3–15% (9), as in our study stones in the gall bladder accounted for 15/119 (12.6%) of the cases followed by cholangitis in 7.5% of the cases.

In our study pancreas was the predominant organ involved. Pancreatitis and pancreatic necrosis together contribute to 31/119 (26%) of cases and bacterial growth was observed in 20/31 (64.5%) of cases. Pancreatic necrosis if infected is an important risk factor and it should be treated by surgery (10). All our cases were treated with surgery and removal of necrosum followed by appropriate antibiotics therapy. In a study by Dionigi R et al. they found that enteric Gram negative bacilli are commonly isolated from acute pancreatitis (11). *E. coli* was isolated in 35.7% of our cases of pancreatic infection.

In a study by Goldstein et al. the most common organism isolated in cases of perforation was *E. coli* (51.72%) followed by *Klebsiella* (29.31%) (2). Similar to our study *E. coli* was predominant organism followed by *Klebsiella*. In the study of Goldstein et al. acute appendicitis was the most common condition requiring emergency surgery (2). In a study by Lahiri et al. from India, the commonest organism isolated was *E. coli* (67.78%) (12) as in our study.

The presence of gallstones within either the gall-bladder or biliary tree is associated with the bacterial colonization of the bile. Similar to our study, *E. coli* was the most common organism isolated in a study conducted by Ballal et al. from India (13).

*E. coli* (62.7%), *K. pneumoniae* (16.7%), and *P. aeruginosa* (5.3%) were the most frequently isolated pathogens in IAI’s from India in a study conducted by Shree et al. (14). As per literature review (1, 2, 13, 14), *E. coli* was the most frequent pathogen associated with IAI which is similar to our study where *E. coli* was isolated from 40.8% of cases.

There is an increase of extended spectrum β-lactamase (ESBL) and multi-drug resistant (MDR) isolates worldwide in the study by Shree et al. (14). Among the intra-operative isolates, ESBL producing *E. coli* isolates comprised 13.7%, while ESBL-positive *K. pneumoniae* isolates represented 18.6% in the study done by CIOWA (7). There was a higher percentage of ESBL producers in *E. coli* (70.6%) and *K. pneumoniae* (72.5%) in our study also which is of increasing concern.

In the study by Goldstein et al, they described the emergence of multidrug-resistant (MDR) Gram-negative bacteria such as *P. aeruginosa, Acinetobacter baumannii, Stenotrophomonas maltophilia* from IAI’s (2). In a study from Greece, 8.8% of intra-abdominal infections is caused by *S. maltophilia* and is intrinsically resistant to multiple classes of antibiotics due to different mechanism of resistance. The drug of choice for infection with this organism is trimethoprim/sulfamethoxazole (15). In our study, we isolated *P. aeruginosa* from one patient with cholangitis, *S. maltophilia* in 3 patients and one *burkholderia cepacia* from a case of pancreatic necrosis. These infections are mostly hospital acquired and are multidrug resistant and require strict infection prevention control measures (14).

*Bacteroides fragilis* accounts for only 0.5% of the normal colonic flora, hence is recognized as the single most important anaerobic pathogen (3). In study of Goldstein et al, *Bacteroides* accounted for 34.6% of the anaerobes isolated from IAI (16). Metronidazole is the choice of therapy for *Bacteroides* (17). We isolated *Bacteroides* from 2 cases of pancreatitis which was low compared to other studies which might have been due to the empirical therapy with metronidazole given to our patients.

*Enterococcus* is the commonest Gram positive bacteria isolated from IAI’s (7). In a study by Marcus et al. they isolated 5 cases of *Enterococcus* and one case of methicillin resistant *S. aureus* (MRSA) from IAI (18). In our study we isolated four cases of *E. faecium* and one case of MRSA. *Enterococcus* does not have a poor prognosis and empiric therapy is not recommended for these organism in community acquired infections (18), whereas it is more prevalent in hospital acquired infections requiring antibiotic therapy (7). In a study from France they found that IAI’s due to MRSA may be due to nasal colonization (19). The source of infection in our case could not be traced.

Our patients were empirically treated with piperacillin-tazobactum, amikacin and metronidazole. As per IDSA guidelines metronidazole is given as an intravenous infusion of 500 mg every 8–12 hr or 1500 mg every 24 h, intravenous piperacillin-tazobactam 3.375 g every 6 hr and amikacin 15–20 mg/kg every 24 hr. The therapy can be discontinued if there is no evidence of infection (20). In patients with post-operative complications and in infections with drug resistant isolates antibiotics were changed based on drug susceptibility reports. Around 27% of *E. coli* and *K. pneumoniae* were MDR isolates and they were sensitive to colistin. All these patients were treated with intravenous colistin 3MU every 8^th^ hourly.

Post-operative complications should be managed intensively with broad spectrum antibiotics for sepsis, leakage, additional surgeries and controlling the source of complication (4). In our study it was less accounting for 10% of the cases.

The rate of mortality was significantly higher in patients with higher degree of pancreatic necrosis from 5–40% as reported by Pal et al. (21). In our study mortality was higher (7/12, 58%) in pancreatic necrosis of all pancreatic involvement, probably due to the higher degree of necrosis in our patients.

The other important cause of mortality in intraabdominal infections is perforative peritonitis (22) and the mortality ranges from 6 and 27% as reported by Oheneh et al. (23) and in small bowel perforations which is common it accounts between 10 and 36.5% according to the report of Bapat et al. (24). Mortality due to perforations was 8/43 (19%) in our study.

As our institute is a tertiary care centre we had complicated cases like pancreatic necrosis and less severe case of acute appendicitis. Multi drug resistant organisms, mostly hospital acquired, were successfully treated as per susceptibility results. All multidrug resistant cases were advised to follow proper infection control measures. There was no significant difference in the organism isolated and organ of infection. Hence empirical treatment can be started based on hospital antibiogram which helps to prevent complications associated with hospital acquired IAI’s.

In conclusion, initial empirical antibiotic therapy should be modified based on the organism isolated and susceptibility results. In addition, patients with intra-abdominal infections should be administered the most potent antibiotics immediately rather than the most commonly used antibiotics. Early diagnosis and appropriate management of the infections will help to prevent the morbidity and mortality associated with these infections.
